# Protection of zero-valent iron nanoparticles against sepsis and septic heart failure

**DOI:** 10.1186/s12951-022-01589-1

**Published:** 2022-09-05

**Authors:** Daquan Wang, Changyu Wang, Zhenxing Liang, Wangrui Lei, Chao Deng, Xiaoli Liu, Shuai Jiang, Yanli Zhu, Shaofei Zhang, Wenwen Yang, Ying Chen, Yao Qiu, Lingjie Meng, Yang Yang

**Affiliations:** 1 Key Laboratory of Resource Biology and Biotechnology in Western China, Ministry of Education, Faculty of Life Sciences and Medicine, Northwest University, 229 Taibai North Road, Xi’an, China; 2 School of Chemistry, Xi’an Jiaotong University, No. 28, West Xianning Road, 710049 Xi’an, Shaanxi China; 3 Xi’an Key Laboratory of Cardiovascular and Cerebrovascular Diseases, Xi’an No. 3 Hospital, School of Life Sciences and Medicine, Northwest University, 10 Fengcheng Three Road, Xi’an, China; 4 Department of Cardiothoracic Surgery, The First Affiliated Hospital of Zhengzhou University, 1 Jianshe East, Zhengzhou, China; 5 Department of Cardiovascular Surgery, The First Affiliated Hospital of Xi’an Jiaotong University, 277 Yanta West Road, Xi’an, China; 6 Department of Hematology, The First Affiliated Hospital of Xi’an Jiaotong University, 277 Yanta West Road, Xi’an, China

**Keywords:** NanoFe, Sepsis, Heart failure

## Abstract

**Background:**

Septic heart failure accounts for high mortality rates globally. With a strong reducing capacity, zero-valent iron nanoparticles (nanoFe) have been applied in many fields. However, the precise roles and mechanisms of nanoFe in septic cardiomyopathy remain unknown.

**Results:**

NanoFe was prepared via the liquid-phase reduction method and functionalized with the biocompatible polymer sodium carboxymethylcellulose (CMC). We then successfully constructed a mouse model of septic myocardial injury by challenging with cecal ligation and puncture (CLP). Our findings demonstrated that nanoFe has a significant protective effect on CLP-induced septic myocardial injury. This may be achieved by attenuating inflammation and oxidative stress, improving mitochondrial function, regulating endoplasmic reticulum stress, and activating the AMPK pathway. The RNA-seq results supported the role of nanoFe treatment in regulating a transcriptional profile consistent with its role in response to sepsis.

**Conclusions:**

The results provide a theoretical basis for the application strategy and combination of nanoFe in sepsis and septic myocardial injury.

**Graphical Abstract:**

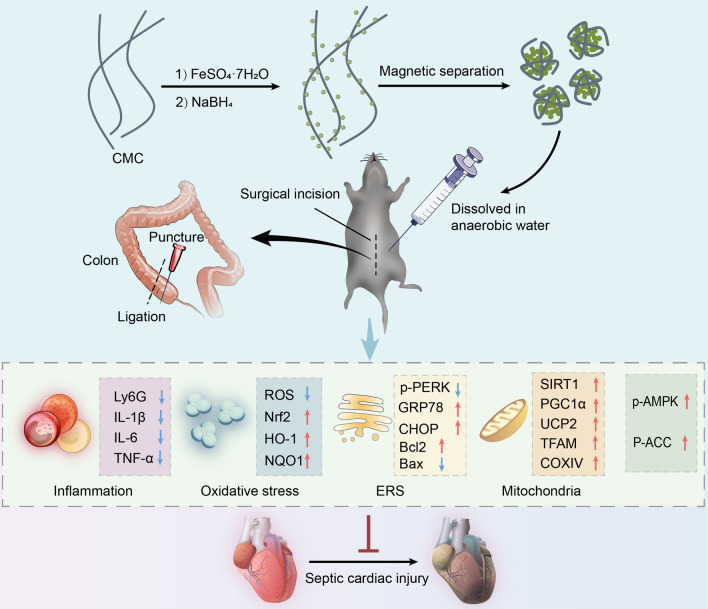

**Supplementary Information:**

The online version contains supplementary material available at 10.1186/s12951-022-01589-1.

## Background

Sepsis is one of the leading causes of death in intensive care units [1]. In recent years, a new definition that sepsis is a life-threatening organ dysfunction caused by a dysregulated host response to infection has been proposed [2]. Remarkably, the main causes of death by coronavirus disease 2019 (COVID-19) include the onset of sepsis. Myocardial injury is one of the essential characteristics of sepsis and can significantly increase the mortality rate of sepsis [3–5]. The pathogenesis of septic myocardial injury involves a complex network, including an exacerbated inflammatory response, excessive production of reactive oxygen species (ROS), and mitochondrial dysfunction. In particular, various reactive species and/or free radicals, such as nitric oxide (NO) and hydrogen peroxide (H_2_O_2_), promote the deterioration of the endothelium and enhance vascular permeability, thereby accelerating the process of septic heart failure [6, 7]. However, due to the rather complicated pathological mechanisms, the clinical methods and drugs for treating septic myocardial injury are limited. It is of great significance to search for successful novel treatments to generate an intense and persistent suppression effect in septic myocardial injury.

Currently, several organic or inorganic nanomaterials are drawing increased attention in a wide variety of biomedical applications, especially as potent antioxidants in treating sepsis, including sustainable nanosheets [8] and ceria nanoparticles [9]. However, safety issues associated with the potential toxicity of nanoparticles and unsatisfactory survival rates during sepsis still remain. Recently, due to the high specific surface area, good bioavailability, and magnetic properties, zero-valent iron nanoparticles (nanoFe) with sizes smaller than 100 nm have been applied in the vast fields of environmental remediation and biomedical applications [10, 11]. Iron is one of the essential micronutrients in the human body; in addition, nanoFe is not mutagenic at low concentrations [12], so nanoFe shows high safety as a drug and has been evaluated in various diseases, including anemia [13] and anticancer therapy [14]. Importantly, nanoFe possesses excellent reducing capacity [15]. Based on these considerations, investigations into the potential of nanoFe as a treatment in sepsis and septic myocardial injury are of great scientific interest.

Herein, the study for the first time was devoted to addressing whether and how nanoFe exerts a protective role in sepsis and septic myocardial injury. First, nanoFe was prepared via the liquid-phase reduction method and functionalized with a biocompatible polymer sodium carboxymethylcellulose (CMC). Then, nanoFe was administered to mice with a cecal ligation and puncture (CLP) model to examine the survival rate, sepsis score, anal temperature, routine blood parameters, blood biochemical parameters, cardiac function indicators, pathological indicators of myocardial tissue injury, the AMPK signaling pathway, and several sepsis-related signaling pathways. RNA-seq detection further illustrated the comprehensive protective mechanisms of nanoFe in this scenario. The results of this study provide a promising therapeutic strategy against sepsis and septic heart failure.

## Results

### Preparation and characterization of nanoFe

**Fig. 1:**
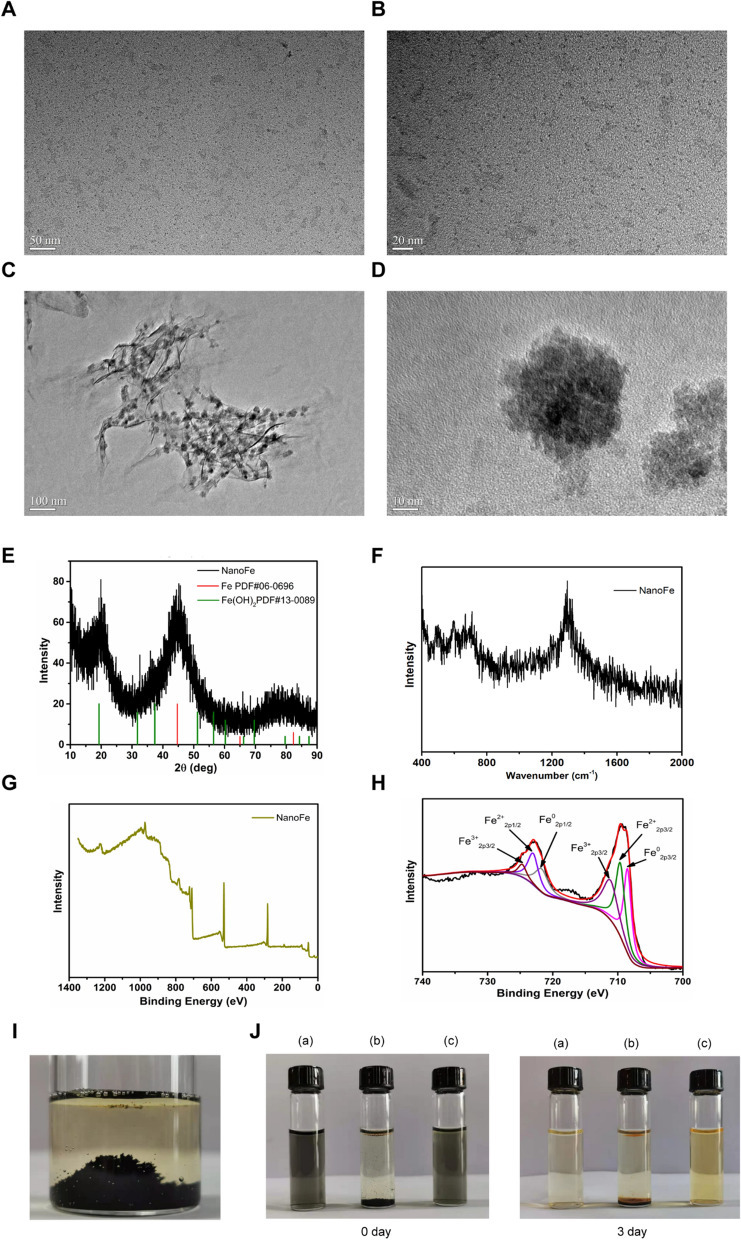
Preparation and spectral characterization of nanoFe**.** TEM images of nanoFe prepared by the reduction of FeSO_4_·7H_2_O using NaBH_4_ in 0.5% CMC solution. **A**, **B** NanoFe in reaction mixture solution. **C**, **D** NanoFe redispersed in 0.5% CMC solution after magnetic separation under ultrasound. **E** Powder XRD spectrum, **F** Raman spectra, **G** XPS Survey spectrum, and **H** Fe 2p3/2 and 2p1/2 spectrum of nanoFe. **I** Hydrogen bubbles were produced by weak hydrolysis of nanoFe in anaerobic water. **J** Photograph of the samples: *a* nanoFe in reaction mixture solution, *b* nanoFe in water after magnetic separation, *c* nanoFe redispersed in 0.5% CMC solution after magnetic separation under ultrasound

NanoFe was prepared as reported [16]. Briefly, FeSO_4_·7H_2_O aqueous solution was mixed with 0.5% sodium CMC aqueous solution, and then ice-cold sodium borohydride (NaBH_4_) aqueous solution was added under mechanical stirring as a reducing agent. The mixture solution instantly turned black, and the iron nanoparticles were washed and collected by magnetic separation after 30 min. The nanomorphology of the iron nanoparticles was studied by high-resolution transmission electron microscopy (HR-TEM) (Fig. 1A–D). As shown in Fig. 1A, B, the primary morphology of nanoFe in the reaction mixture took on tiny nanodots with a uniform diameter of approximately 1.2 nm. After the iron nanoparticles were collected by magnetic separation and redispersed in CMC solution, the iron nanoparticles clustered together due to the stronger residual magnetic field, and the iron cluster showed an increased size of approximately 20–40 nm (Fig. 1C, D).

Structure and element measurements, including powder X-ray diffraction (XRD), Raman spectroscopy, and X-ray photoelectron spectroscopy (XPS), were carried out to confirm the main compositions of nanoFe (Fig. 1E–G). According to the standard PDF card of iron and its compounds, the strong peak at 44.7° should be assigned to α-Fe (PDF#06-0696), and the peak at 19.3° should be assigned to Fe(OH)_2_ (PDF#13-0089), suggesting that the main component of the prepared nanoFe was iron and a small amount of Fe(OH)_2,_ which should come from the weak hydrolysis on the surface of nanoFe in water. Indeed, the fresh nanoFe in oxygen-free water continued to produce small bubbles, meaning that the nanoFe reacted with H_2_O and generated Fe(OH)_2_ and H_2_. In the 400–800 cm^−1^ range of Raman spectroscopy (Fig. 1F), the identifiable peaks also confirmed the presence of iron in the oxygen state on the surface of iron nanoparticles. For example, 595 cm^−1^ corresponds to iosiderite (FeO), 535 cm^−1^ and 650 cm^−1^ correspond to lepidocrocite (γ-FeOOH), and 514 cm^−1^ and 667 cm^−1^ correspond to maghemite (γ-Fe_2_O_3_) [17]. XPS was further used to confirm the presence and content of Fe(II) and Fe(III) on the surface of nanoFe (Fig. 1G, H). The peak area ratio of the three valence states is 33.4:35.5:31.1 (Additional file 1: Table S1), indicating the relatively high content oxidation state of Fe on the surface of the nanoFe, probably due to the rapid oxidation of Fe(II) to Fe(III) when it is exposed to oxygen. Figure 1I, J shows the oxidation of nanoFe in water. The dispersed nanoFe in the CMC solution showed a black–green color and changed to yellow, which is the characteristic color of Fe(III), after remaining still for 3 d.

### Acute toxicity of nanoFe

The safety of nanoFe was evaluated by an acute oral toxicity experiment. After food deprivation for 12 h, female and male mice (10 each) were intraperitoneally injected with 300 mg/kg nanoFe 3 times at an interval of 6 h. Control female and male mice (2 each) were injected intraperitoneally with the same volume of deoxygenated water in the same manner. Food and water were provided 4 h later. Their movement, bite and sup, feces mortality, and body weight were monitored continuously for 14 d. As shown in Additional file 1: Table S2 and Additional file 1: Fig. S1A, no death and no abnormal clinical signs, related to appetite, diarrhea, hypnosia, mortality and weight change, were observed during an investigation period of 14 d. In addition, hematoxylin–eosin (H&E) staining revealed no apparent morphological change or injury of cardiac and renal tissue in control mice and nanoFe-treated mice, while the slight damage was observed in the liver (Additional file 1: Fig. S1B). Subsequently, we observed the effect of 20 mg/kg nanoFe (3 times at an interval of 1 d, the maxium protective dosage used in this study) on tissue structure of male mice, and found almost no toxicity on the the heart, liver, and kidney (Additional file 1: Fig. S1C). These results suggested that a certain dose of nanoFe caused no toxic effects in mice.

### Protective effects of nanoFe treatment against sepsis in mice

**Fig. 2:**
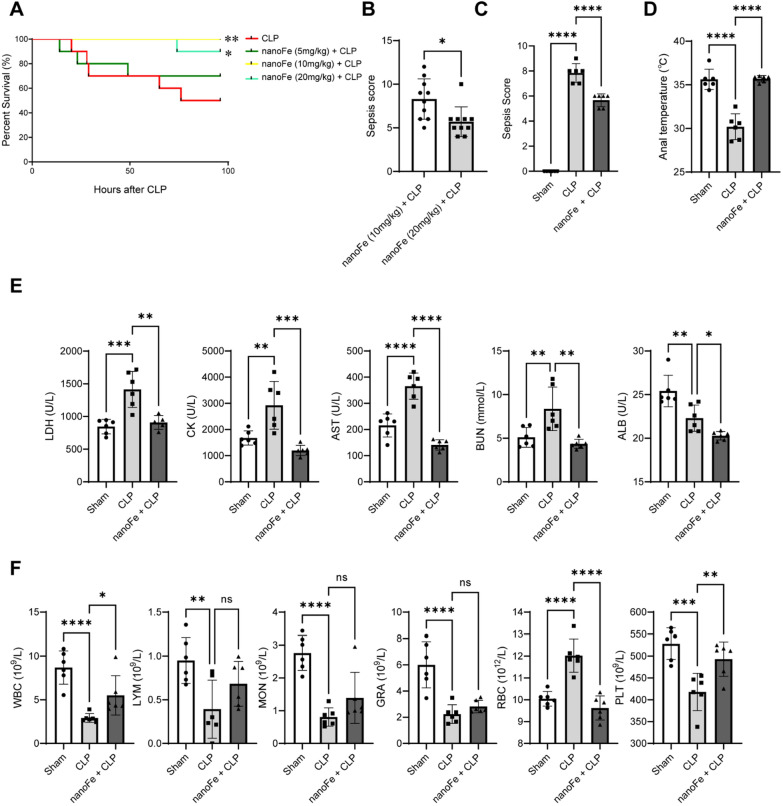
Protective effects of nanoFe treatment in septic mice. **A** Survival curves indicating the survival of mice after treatment of different concentrations of nanoFe (5, 10, and 20 mg/kg). Mortality was observed within 96 h. (n = 10 for each group). **B** The sepsis score recorded after treatment with nanoFe (10 mg/kg) and nanoFe (20 mg/kg) (n = 10 for each group). **C** The sepsis score of Sham, CLP, and nanoFe (20 mg/kg) + CLP mice (n = 6 for each group). **D** The anal temperature (n = 6 for each group). **E** Blood biochemical parameters (n = 6 for each group). **F** Blood routine parameters (n = 6 for each group). ^*^P < 0.05, ^**^P < 0.01, ^***^P < 0.001, ^****^P < 0.0001 *vs.* Sham or *vs.* CLP (panel **A** and **C**-**F**) or *vs*. nanoFe (10 mg/kg) + CLP (panel **B**); ns, non-significant. Statistical analysis of **B** was performed using t-test. Statistical analysis of the other data was used ANOVA

The CLP method is one of the classical models since it provides a better representation of the pathophysiology of human sepsis [18]. Hence, the CLP-induced sepsis model was first successfully constructed for subsequent experiments (Additional file 1: Fig. S2A). Then, mice were given different concentrations of nanoFe (5, 10, and 20 mg/kg) to observe the survival rate within 96 h post-CLP. As shown in Fig. 2A, approximately 50% of the CLP mice died. However, after pretreatment with different concentrations of nanoFe, the survival rate in the nanoFe + CLP group increased significantly to more than 60%, among which the survival rate of mice treated with the 10 mg/kg and 20 mg/kg nanoFe groups was higher (vs. the CLP group, *P* < 0.05). Additionally, the sepsis score of 20 mg/kg nanoFe was lower than that of 10 mg/kg nanoFe (Fig. 2B-C, *P* < 0.05). Therefore, 20 mg/kg nanoFe was selected for further functional experiments. As shown in Fig. 2D, nanoFe could also elevate the anal temperature of CLP mice (*P* < 0.05). Routine blood parameters showed that, compared with the CLP group, nanoFe treatment markedly increased the numbers of white blood cells (WBCs) and platelets (PLTs) while decreasing the levels of red blood cells (RBCs) (Fig. 2F, *P* < 0.05). The results suggested the protective roles of nanoFe in sepsis.

### Protective effects of nanoFe treatment against septic myocardial injury in mice

**Fig. 3:**
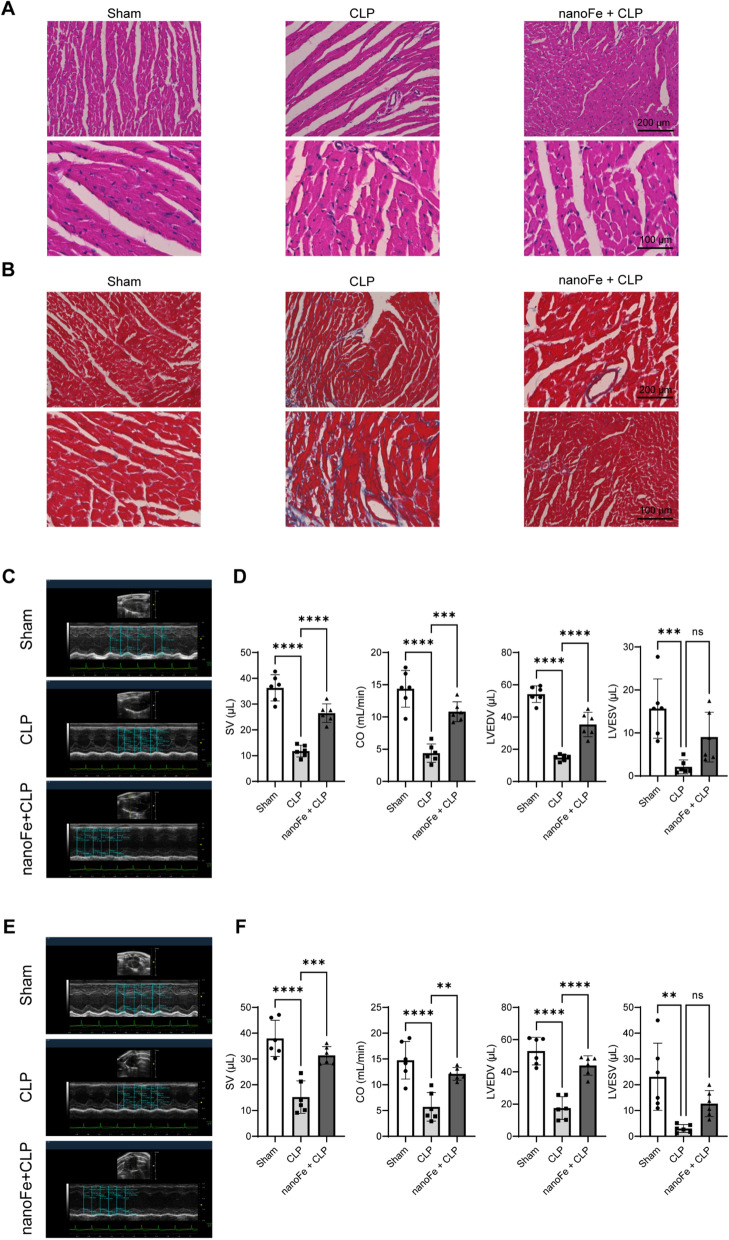
Protective effects of nanoFe treatment against septic myocardial injury in mice. **A** Representative images of H&E staining of mouse heart tissues. **B** Representative images of Masson’s trichrome staining of mouse heart tissues. **C** Representative echocardiography images in the long axis. **D** SV, CO, LVEDV, and LVESV statistical graphs of the long axis. **E** Representative echocardiography images in the short axis. **F** SV, CO, LVEDV, and LVESV statistical graphs of the short axis. ^*^P < 0.05, ^**^P < 0.01, ^***^P < 0.001, ^****^P < 0.0001 vs. Sham or *vs.* CLP; ns, non-significant. n = 6 for each group. Statistical analysis of the data was used ANOVA

Then, the myocardial structure was examined, and cardiac function was assessed in septic mice. H&E staining revealed more severe myocardial tissue injury in septic mice, as evidenced by more myocardial fibers, aggravated interstitial edema, and destroyed cellular integrity (vs. the sham group, Fig. 3A). Consistently, Masson’s trichrome staining showed the significantly elevated cardiac fibrosis in CLP mice (vs. the sham group, Fig. 3B). The echocardiographic analysis revealed a significant reduction in cardiac output (CO), stroke volume (SV), left ventricular diastolic volume (LVEDV), and left ventricular systolic volume (LVESV) in CLP mice compared to Sham mice (Fig. 3C–F,* P* < 0.05). Additional echocardiographic data are also presented in Additional file 1: Fig. S3. Similarly, the levels of serum biomarkers of myocardial injury, such as lactic dehydrogenase (LDH), creatine kinase (CK), and aspartate aminotransferase (AST), rose significantly after CLP injury (vs. the sham group, Fig. 2E,* P* < 0.05). However, the changes in cardiac structure, the decrease in cardiac function indicators, and the increase in blood biochemical parameters induced by CLP were all significantly reversed in nanoFe-treated mice (vs. the CLP group, Figs. 2E and 3, *P* < 0.05). The above data suggested that nanoFe treatment exerted protective effects against myocardial injury both structurally and functionally in septic mice.

### Effects of nanoFe treatment on the myocardial inflammatory response in septic mice

**Fig. 4:**
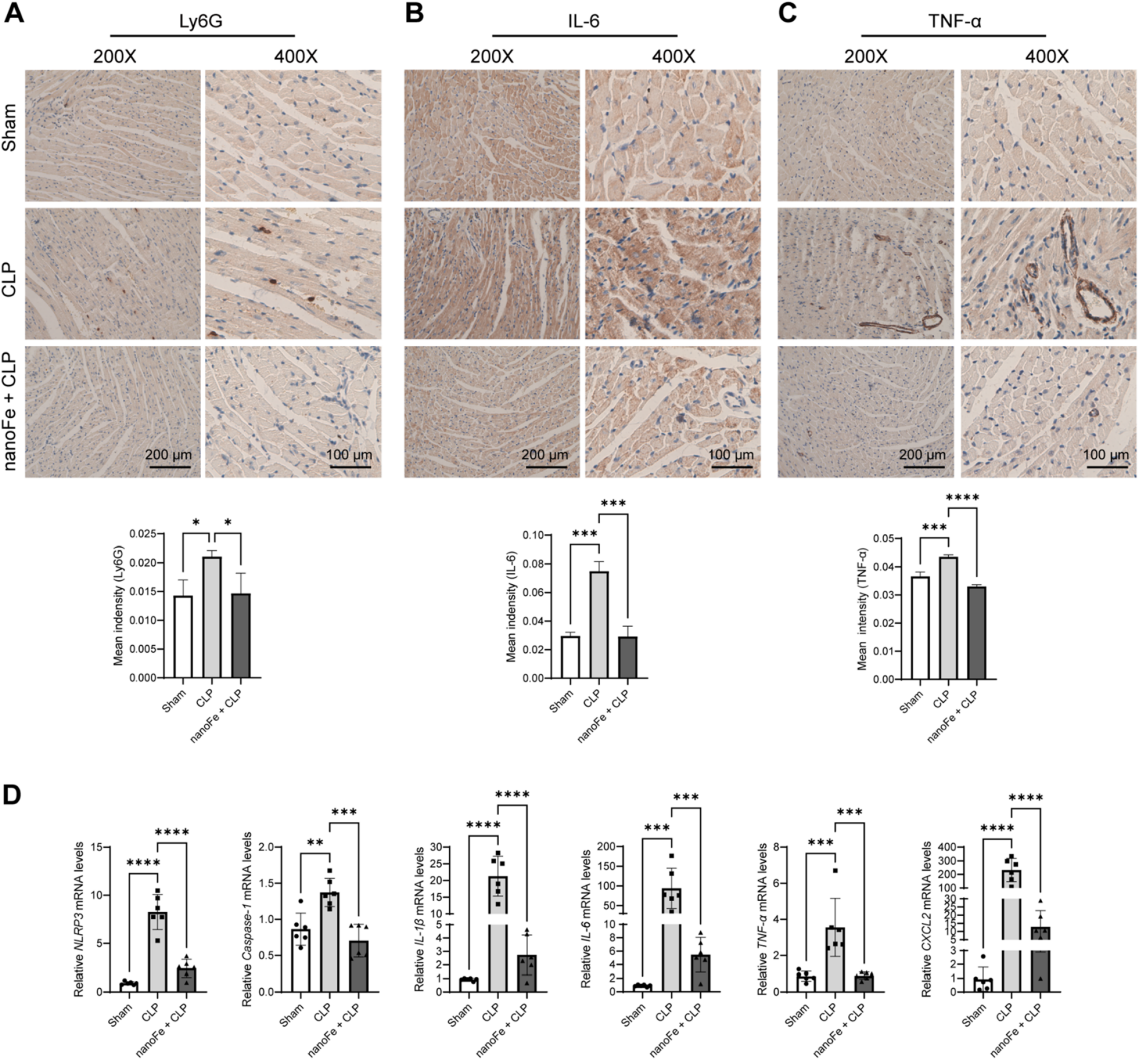
Protective effects of nanoFe treatment on myocardial inflammatory response in septic mice. Representative photographs of IHC staining of **A** (Ly6G), **B** (IL-6), and **C** (TNF-α). **D,** qRT-PCR analysis of myocardial NLRP3, Caspase-1, IL-1β, IL-6, TNF-α, and CXCL2. ^*^P < 0.05, ^**^P < 0.01, ^***^P < 0.001, ^****^P < 0.0001 *vs.* Sham or *vs.* CLP; ns, non-significant. n = 6 for each group. Statistical analysis of the data was used ANOVA

Inflammation is one of the major characteristics of sepsis and is involved in detrimental pathways activated during sepsis, eventually leading to cardiac dysfunction and death [19]. Ly6G is a marker of macrophage and neutrophil accumulation. As shown in Fig. 4A, CLP led to increased myocardial contents of Ly6G in the heart, but the extent was much less in the nanoFe + CLP group (vs. the CLP group, *P* < 0.05). Similarly, the increased levels of the inflammatory factors interleukin (IL)-6 and tumor necrosis factor-α (TNF-α) were also ameliorated by nanoFe, as demonstrated by immunohistochemistry (IHC) staining and quantitative real-time PCR (qRT-PCR) (vs. the CLP group, Fig. 4B–D,* P* < 0.05). In addition, nanoFe significantly reduced the mRNA levels of nucleotide-binding domain leucine-rich repeat and pyrin domain containing receptor 3 (NLRP3), cysteinyl aspartate specific proteinase (Caspase-1), IL-1β, and chemokine CXCL2 (vs. the CLP group, Fig. 4D,* P* < 0.05). These data together confirmed that nanoFe treatment alleviated CLP-induced myocardial inflammation.

### Effects of nanoFe treatment on myocardial oxidative stress and antioxidative signaling in septic mice

**Fig. 5:**
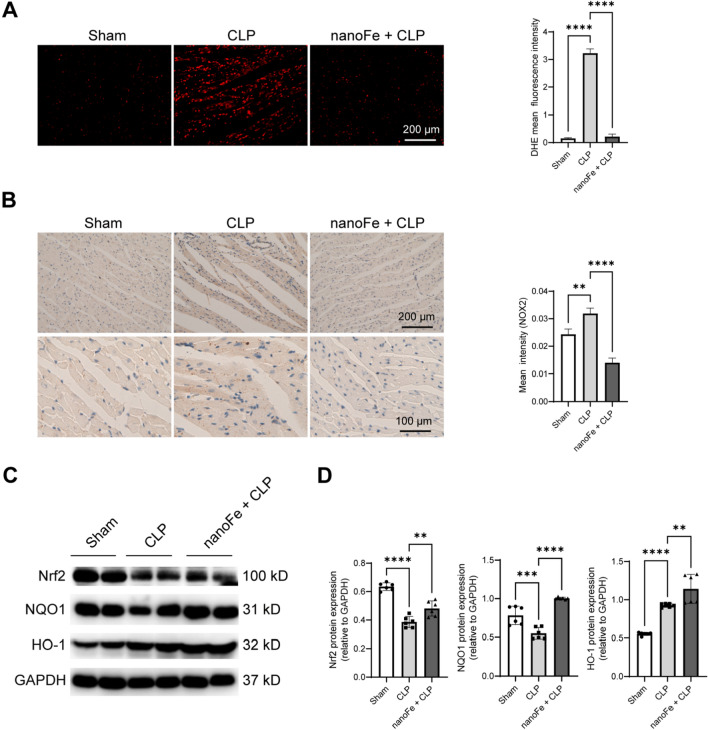
Protective effects of nanoFe treatment on myocardial oxidative stress in septic mice. **A** Representative photographs of in DHE of mouse heart tissues. **B** Representative photographs of IHC staining of NOX2. **C** Representative Western blot images of Nrf2, NQO1, and HO-1. **D** Quantitative analysis of **C** was determined with GAPDH for normalization. Statistical analysis of the data was used ANOVA

The study then evaluated myocardial ROS generation by in situ dihydroethidium (DHE) staining and found that ROS levels increased significantly in CLP mice, while a significant decrease was observed in nanoFe-treated septic mice (vs. the CLP group, Fig. 5A,* P* < 0.05). The IHC staining of NOX2 (ROS marker) indicated similar changes (vs. the CLP group, Fig. 5B,* P* < 0.05). In addition, the presence of antioxidative mediators, including nuclear factor-related Factor 2 (Nrf2), NAD(P)H dehydrogenase quinone-1 (NQO1), and heme oxygenase-1 (HO-1), was detected by Western blot. As shown in Fig. 5C, D, myocardial Nrf2 and NQO1 showed a declining pattern after CLP, while nanoFe reversed this change and enhanced the expression of HO-1 (vs. the CLP group, *P* < 0.05). These results suggest that nanoFe treatment mitigated oxidative stress during septic myocardial injury.

### Effects of nanoFe treatment on myocardial endoplasmic reticulum stress (ERS), apoptosis, mitochondrial dysfunction, and the AMPK signaling pathway in septic mice

To elucidate the molecular mechanisms of the protective effects that nanoFe treatment had on septic myocardial injury, the alterations of other essential sepsis-related pathophysiological processes, such as ERS, mitochondrial dysfunction, and apoptosis, were further investigated in heart tissues from CLP mice.

**Fig. 6:**
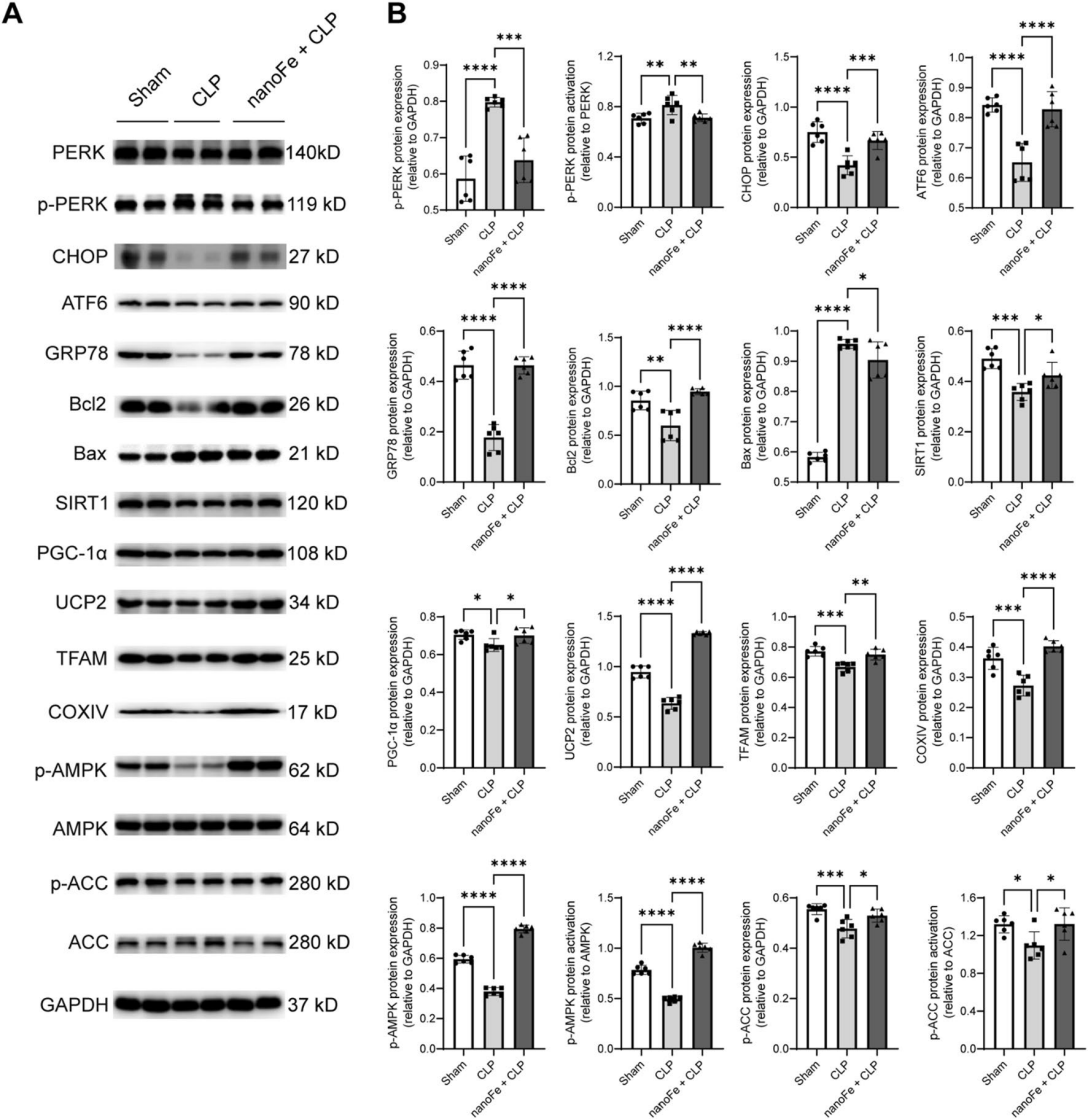
Effects of nanoFe treatment on myocardial ERS, mitochondrial dysfunction, and apoptosis in septic mice. **A** Representative Western blot images of PERK, p-PERK, CHOP, ATF6, GRP78, Bcl2, Bax, SIRT1, PGC-1α, UCP2, TFAM, COXIV, p-AMPK, AMPK, p-ACC, and ACC. **B** Quantitative analysis of **A** was determined with GAPDH for normalization. ^*^P < 0.05, ^**^P < 0.01, ^***^P < 0.001, ^****^P < 0.0001 *vs.* Sham or *vs.* CLP; ns, non-significant. n = 6 for each group. Statistical analysis of the data was used ANOVA

Convincing evidence has suggested the role of ERS in septic heart failure [20]. The levels of ERS-related proteins, including PKR-like endoplasmic reticulum kinase (PERK), p-PERK, C/EBP homologous protein (CHOP), activating transcription Factor 6 (ATF6), and glucose-regulated protein 78 (GRP78), in the myocardium of septic mice were evaluated. Western blot results demonstrated that CLP injury markedly elevated the levels of p-PERK and p-PERK/PERK, while decreased the levels of CHOP, ATF6, and GRP78. In contrast, these effects were blunted by nanoFe treatment, indicating the regulation of ERS pathways may be involved in the protection of nanoFe against septic myocardial injury (vs. the CLP group, Fig. 6A, B,* P* < 0.05). Regarding myocardial apoptosis, compared with the CLP group, the expression of proapoptotic Bcl-2-associated X (Bax) declined significantly while the levels of the prosurvival B-cell lymphoma-2 (Bcl2) rose in nanoFe-treated septic mice (Fig. 6A, B, *P* < 0.05).

**Fig. 7:**
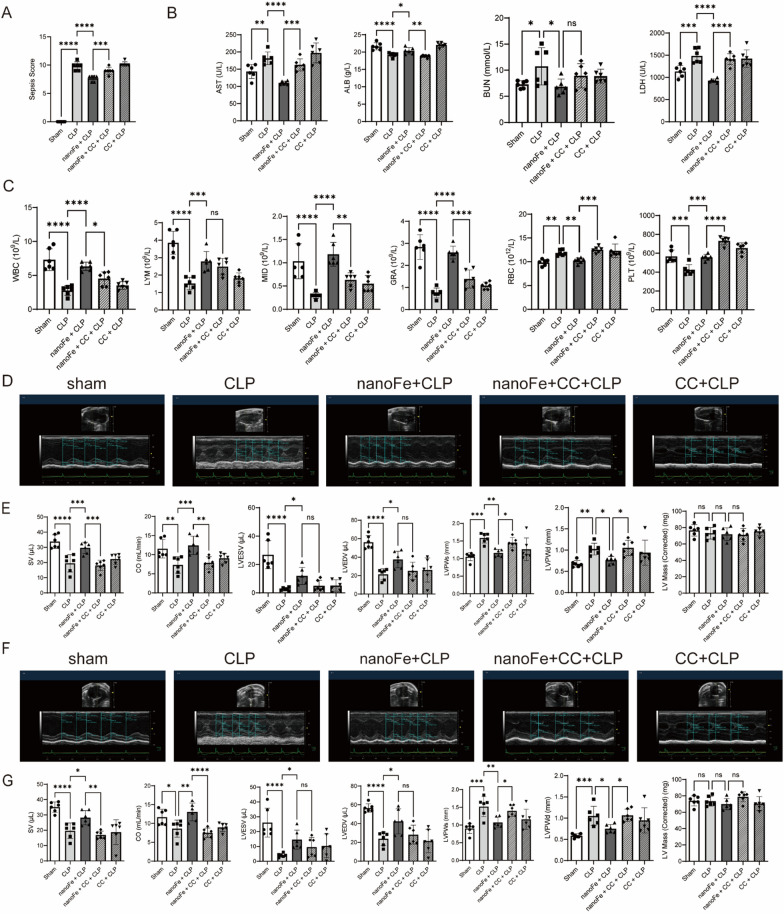
Effects of CC on the protective role of nanoFe in septic mice.** A** The sepsis score. **B** Blood biochemical parameters. **C** Blood routine parameters. **D** Representative echocardiography images of the long axis. **E** SV, CO, LVEDV, LVESV, LVPWs, LVPWd, and LV Mass statistical graphs of the long axis. **F** Representative echocardiography images of the short axis. **G** SV, CO, LVEDV, LVESV, LVPWs, LVPWd, and LV Mass statistical graphs of the short axis. *P < 0.05, **P < 0.01, ***P < 0.001, ****P < 0.0001 vs. Sham or vs. CLP or nanoFe + CLP; ns, non-significant. n = 6 for each group. Statistical analysis of data was performed using ANOVA

Mitochondrial biogenesis regulatory programs, such as the silent information regulator 1/peroxisome proliferator-activated receptor coactivator-1α/mitochondrial transcription Factor A (SIRT1/PGC-1α/TFAM) pathway, uncoupling protein 2 (UCP2), and cytochrome oxidase subunit IV (COXIV), can improve mitochondrial biogenesis and cardiac dysfunction during sepsis [21, 22]. This study proved that the levels of SIRT1, PGC-1α, UCP2, TFAM, and COXIV all decreased in septic mice, the effect of which was reconciled by nanoFe treatment (vs. the CLP group, Fig. 7A, B *P* < 0.05), indicating the positive role of nanoFe in improving mitochondrial biogenesis during sepsis.

The AMP-activated protein kinase (AMPK)/acetyl-CoA carboxylase (ACC) signaling pathway is a classic myocardial protection pathway of energy metabolism. However, sepsis can cause a rapid decrease in the phosphorylation and activity of AMPK and ACC [23]. Consistently, the results revealed a significant decline in AMPK and ACC phosphorylation levels in septic mice, while the reduction in those levels was reversed by nanoFe treatment (vs. the CLP group, Fig. 6A, B, *P* < 0.05). These results suggest that the AMPK/ACC signaling pathway participated in the cardioprotection of nanoFe during sepsis.

### AMPK inhibition worsened CLP-induced myocardial injury

To further identify whether AMPK signaling is more important as the major mechanism of nanoFe modulation, mice were pretreated with Compound C (CC, an AMPK inhibitor) and nanoFe. As shown in Fig. 7A, CC markedly reversed the nanoFe-induced decrease in the sepsis score (vs. the nanoFe + CLP group, *P* < 0.05). NanoFe-mediated downregulation of AST, LDH, and RBC and upregulation of ALB, WBC, middle cells (MID), GRA, and PLT were also reversed by CC treatment (vs. the nanoFe + CLP group, Fig. 7B, C,* P* < 0.05). In particular, CC reversed the nanoFe-induced increase in SV and CO and decrease in LVPWs and LVPWd in CLP mice (vs. the nanoFe + CLP group, Fig. 7D–G, * P* < 0.05). Taken together, these data suggest that CC nullified nanoFe-offered cardioprotection against CLP injury and AMPK plays a key role in nanoFe protection against septic myocardial injury.

### RNA-seq illustrated the comprehensive transcriptome regulated by nanoFe in septic mice

**Fig. 8:**
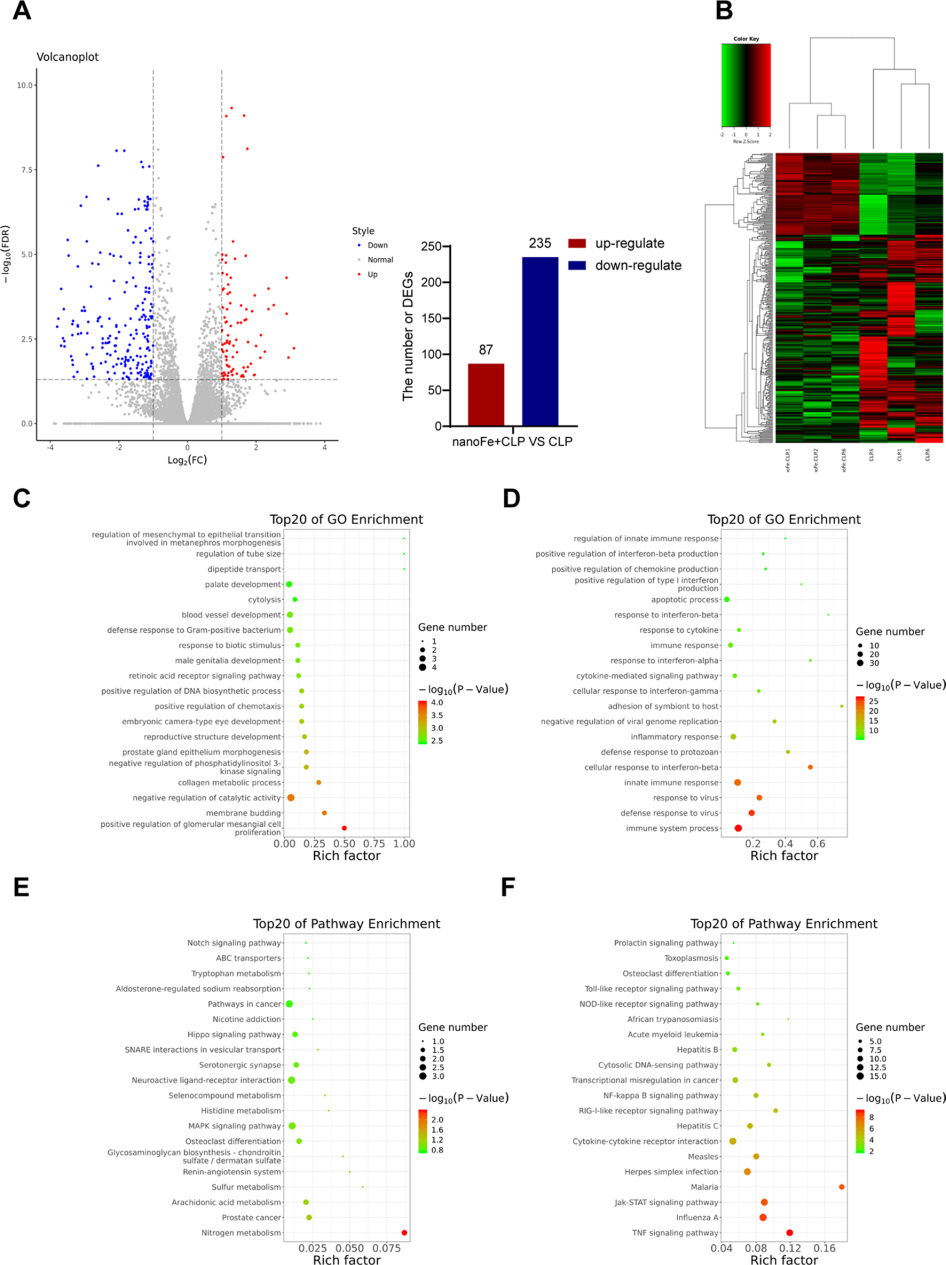
RNA sequencing-identified transcriptome regulated by nanoFe in septic mice. **A** A volcano plot displayed differentially expressed mRNAs. The blue and red parts indicated > twofold decreased or increased expression of the dysregulated mRNAs in cardiac tissues, respectively (*P* < 0.05). **B** Cluster analysis of differential genes in each group. **C** Top 20 GO enrichment for the up-regulated gene categories between the CLP group and the nanoFe + CLP group (*P* < 0.05). **D** Top 20 GO enrichment for the downregulated gene categories between the CLP group and the nanoFe + CLP group. **E** Top 20 KEGG enrichment for up-regulated pathways between the CLP group and the nanoFe + CLP group (*P* < 0.05). **F** Top 20 KEGG enrichment for downregulated pathways between the CLP group and the nanoFe + CLP group (*P* < 0.05)

To obtain comprehensive insight into the pathological regulatory mechanism of nanoFe on CLP injury, a transcriptomic analysis in septic hearts from mice was performed. Biological replicates clustered together among all groups, while each experimental group exhibited a distinct transcriptome profile. RNA-seq revealed 87 upregulated genes and 235 downregulated genes in the nanoFe + CLP group, suggesting that the alteration of these genes is dependent on nanoFe treatment (Fig. 8A, B).

**Fig. 9:**
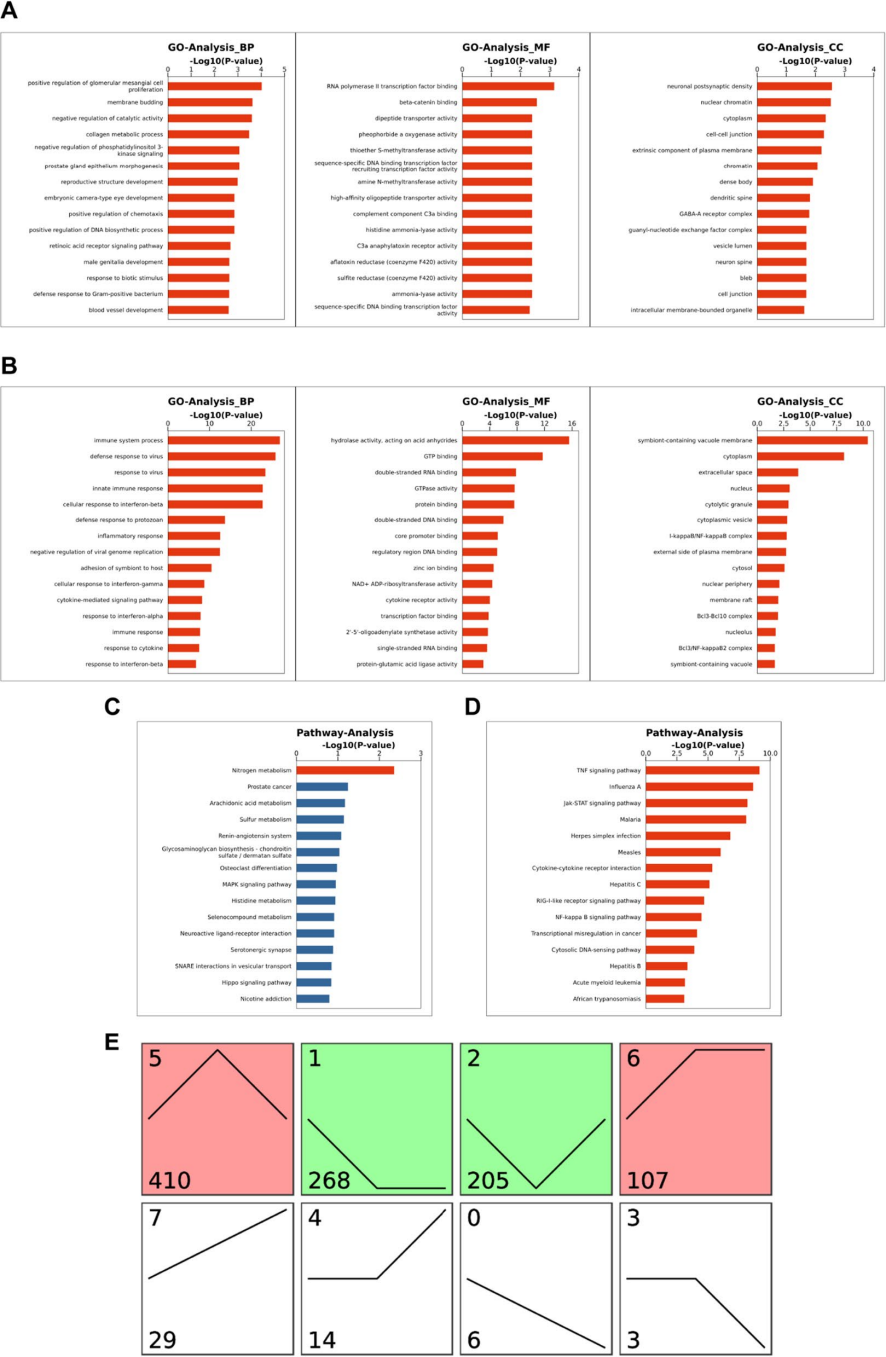
Additional data of RNA sequencing between the CLP group and the nanoFe + CLP group. **A** GO analysis of the up-regulated gene categories (*P* < 0.05). **B** GO analysis of the downregulated gene categories (*P* < 0.05). **C** KEGG pathway analysis of up-regulated pathways (*P* < 0.05). **D** KEGG pathway analysis of downregulated pathways (*P* < 0.05). **E** Expression profiles in color indicated significant ones (*P* < 0.05). Red indicated up-regulated and green indicated downregulated. Profile number (up left), gene number (bottom left), and trend (line) in each profile were also labeled

In addition, gene ontology (GO) was performed to determine functional changes during septic myocardial injury after nanoFe treatment. Upregulated genes in the nanoFe + CLP group included genes involved in positive regulation of glomerular mesangial cell proliferation and membrane budding about the biological process (BP); RNA polymerase II transcription factor binding and beta-catenin binding about the biological molecular function (MF); neuronal postsynaptic density and nuclear chromatin about the cellular component (CC) (Figs. 8C and 9A). Downregulated gene categories in the nanoFe + CLP group included immune system process, defense response to virus, and innate immune response (BP); hydrolase activity, acting on acid anhydrides and GTP binding (MF); symbiont-containing vacuole membrane and cytoplasm (CC) (Figs. 8D and 9B).

Kyoto Encyclopedia of Genes and Genomes (KEGG) pathway analysis further revealed that nanoFe upregulated the pathways of nitrogen metabolism (Figs. 8E and 9C) and simultaneously downregulated the pathways pertinent to the TNF signaling pathway, influenza A, JAK/STAT signaling pathway, and malaria (Figs. 8F and 9D).

All significant mRNAs were further divided into 8 different patterns of dynamic expression. The number of mRNAs and *P* values for each group were calculated, among which four patterns were significant: pattern 5 (upregulated after CLP but downregulated after treatment with nanoFe), pattern 2 (downregulated after CLP but upregulated after treatment with nanoFe), pattern 1 (downregulated after CLP and remained after treatment with nanoFe), and pattern 6 (upregulated after CLP and remained constant after treatment with nanoFe) (Fig. 9E). The results further verify that nanoFe treatment exerted a significant influence on the transcriptome of septic myocardial injury.

## Discussion

Heart failure is a common complication of sepsis, often with a high mortality rate [24]. Increased awareness has been raised for the pathophysiology of sepsis. However, the incidence and mortality of sepsis in clinical treatment are still worrisome. Therefore, sepsis remains a formidable challenge for clinicians and basic researchers. NanoFe holds excellent potential for biomedical, clinical, and environmental applications due to its high specific surface area, good bioavailability, and magnetic properties [25]. Previous studies have evaluated the potential therapeutic application of nanoFe in iron-deficient anemia [13] and cancer [26], hinting its high safety. Shen et al. reported a novel β-lactoglobulin fibril-Fe nanoparticle hybrid material for use in Fe fortification and treatment for anemia [13]. Yu et al. showed that polydopamine-modified nanoFe displayed excellent biocompatibility and tumoricidal ability in breast cancer [26]. In addition, its excellent performance in capturing ROS [10] and antibacterial activity [25] confer nanoFe with therapeutic potential in treating infection-related diseases. Anbouhi et al. showed that nanoFe provides a potential antibacterial effect against *E. coli*, *P. aeruginosa*, and *S. aureus* [27]. Hence, it is reasonable to speculate that nanoFe might be a promising therapeutic strategy against sepsis and sepsis-induced heart failure. Herein, the study developed an effective nanotherapeutic based on nanoFe via a liquid-phase reduction method. To confirm these assumptions, the effect of nanoFe treatment on CLP-injured mice was observed first. As anticipated, nanoFe increased the survival rate, accompanied by the reduced sepsis score and increased anal temperature. Additionally, sepsis led to an increase in serum myocardial damage biomarkers (LDH, CK, and AST) and a decrease in cardiac function indicators (SV, CO, LVEDV, and LVESV). NanoFe treatment shows remarkable improvement in these parameters, hinting the powerful protective effect of nanoFe.

Sepsis is characterized by an overwhelming systemic proinflammatory response to infection and is partly attributable to a “cytokine storm”, such as IL-1β, IL-6, and TNF-α [19]. Inflammatory cells are recruited to the site of injury within the early stage of sepsis, which leads to endothelial dysfunction, vascular plugging, and the release of proinflammatory factors, degradative enzymes, and ROS [28]. Macrophages are the primary inflammatory cells in the lesion region and provide strong proinflammatory signals in the early stage [29]. In addition, neutrophils are recruited to lesion sites and release chemotactic factors to eradicate microbes [30]. Ly6G is the major protein released by degranulated neutrophils and is often used as a marker of neutrophil activation in these conditions [31, 32]. In this work, the beneficial effect of nanoFe on the myocardial inflammatory response manifested as decreased protein levels of Ly6G, IL-6, and TNF-α and markedly decreased mRNA levels of NLRP3, caspase 1, IL-1β, IL-6, TNF-α, and CXCL2. These results indicates the powerful anti-inflammatory ability of nanoFe against sepsis and septic myocardial injury, which is also consistent with the potential antimicrobial effects of nanoFe reported in a previous study [27].

Excessive ROS, produced either by the myocardium itself or by infiltrating inflammatory cells, modify a cascade of intracellular events and provoke severe intracellular oxidative stress [33]. NOX2 can induce ROS and is responsible for sepsis-induced oxidative stress [34]. In addition, Nrf2 is a basic leucine zipper transcription factor that induces antioxidant enzymes, such as HO-1 and NQO1. Developing an efficient antioxidant therapy for sepsis by scavenging ROS or targeting the antioxidant systems is imperative. Fe-based biocatalysts may represent an efficient antioxidant therapy for sepsis due to its great electronegativity and large specific surface area [35]. The standard electrode potential E0 [Fe(II)/Fe(0)] of nanoFe is − 0.44 V, which has strong reducibility and can be used as an antioxidant to play the role of electron donor, thereby transferring electrons to water or oxidizing substances. In vivo, ROS (such as hydroxyl radical, singlet oxygen, hydrogen peroxide and superoxide anion) are extremely oxidizing compared with water, and thus readily accept electrons from iron nanoparticles to be quenched. Similarly, Yang et al. found that magnesium with strong reducing property induces mitochondrial dysfunction and intracellular redox homeostasis destruction by producing hydrogen [36]. Zhu et al. simply mixed gallic acid and Fe3^+^ to form coordination compound nanoparticles (GANPs) and demonstrated that GANPs possesses a high ability to scavenge ROS based on their reducing capacity [37]. Their studies showed that the presence of Fe3^+^ did not affect the reducibility of polyphenols, that is, its ability to quench ROS. We also shown in this study, nanoFe not only reduced the levels of CLP-induced oxidative stress indicators, such as ROS and NOX2, but also increased the expression of antioxidative molecules (Nrf2, NQO1, and HO-1) in the heart tissues. This indicates that the Nrf2/NQO1 (or HO-1) axis may underlie the antioxidative effect of nanoFe during septic heart failure.

Endoplasmic reticulum dysfunction is associated with increased levels of misfolded proteins and increased cellular stress [38]. During sepsis, the production of proinflammatory cytokines and depletion of oxygen can induce the adaptive and proapoptotic pathways of the unfolded protein response (UPR) in the heart, aimed at restoring endoplasmic reticulum homeostasis [39]. The UPR is mainly mediated by three endoplasmic reticulum transmembrane proteins in eukaryotic cells, including PERK, inositol-requiring enzyme-1 (IRE1), and ATF6. Under homeostatic conditions, ERS sensor proteins are bound to GRP78, while the separation of GRP78 from these molecules promotes the correct folding of proteins. However, when the UPR fails to resolve stress, ERS is prolonged, and the persistent activation of other signals (such as prosurvival Bcl2 and proapoptotic Bax) can lead to apoptosis pathways. The current study also observed that nanoFe promoted the expression of GRP78, CHOP, ATF6, and Bcl2 while suppressed the levels of p-PERK, PERK/p-PERK, and Bax. These data imply that the regulation of ERS pathways and inhibition of apoptosis pathways induced by nanoFe are involved in protective roles against septic myocardial injury.

Mitochondrial dysfunction is intrinsically involved in the pathogenesis of sepsis-induced multiple organ failure, particularly in the heart [40]. SIRT1 can activate several transcription factors, such as PGC-1α, resulting in enhanced mitochondrial biogenesis under energy deficiency during sepsis [41]. TFAM and UCP2, regulated by PGC-1α, are critical molecular conductors of mitochondrial biogenesis. In addition, the cytochrome oxidase COXIV can also be activated by upregulation of TFAM to provide electrons for the respiratory chain [42]. Here, nanoFe treatment markedly improved mitochondrial biogenesis, manifested as increased expression of all these proteins. Notably, COXIV and UCP2 regulate not only mitochondrial ATP production but also the generation of ROS.

AMPK is a classical cellular energy sensor that rewires metabolism and maintains redox balance. Previous reports proposed that AMPK activity responds to redox changes by ROS acting on redox-sensitive cysteine residues of the AMPK α subunit [43, 44]. The action of AMPK regulating cellular mechanisms is highly associated with septic myocardial injury [45, 46]. Notably, AMPK activators have been employed as a new approach to alleviating inflammation in ROS-related diseases, including septic-induced injury and cardiovascular diseases [47–49]. In the present study, the results also revealed that nanoFe treatment could significantly promote the activation of AMPK and AMPK-dependent phosphorylation of ACC, implicating the protective role of AMPK induced by nanoFe in sepsis and myocardial sepsis. Further studies have shown that CC reversed the cardioprotection of nanoFe against sepsis, as evidenced by the deterioration of the sepsis score, routine blood parameters, blood biochemical parameters, and cardiac function parameters. Therefore, AMPK might play a key role in nanoFe protection against septic myocardial injury. However, the explicit regulatory crosstalk between these pathways in cardioprotection of nanoFe against sepsis requires further investigation.

As discussed above, the cardioprotection mechanism of nanoFe in sepsis is complicated. We also performed transcriptome sequencing to further clarify the cardioprotective targets of nanoFe. Treatment with nanoFe significantly altered the expression profile of cardiomyocytes. Furthermore, GO profiling and KEGG revealed that these molecules are functionally engaged in not only multiple cellular components but also molecular functions and biological processes. In particular, the myocardial protective mechanisms of nanoFe are involved in the downregulated pathways pertinent to immune system processes, defense responses to viruses, and innate immune responses, the TNF pathway, and the JAK/STAT pathway, which also strongly proves the anti-inflammatory activity of nanoFe in septic myocardial injury. In future research, we will further verify the signaling pathways obtained by RNA-seq and clarify the specific mechanisms of the biological processes.

Although nanoFe is widely used in soil and groundwater remediation, pharmaceuticals, medicine, food production, and other manufacturing processes due to its low toxicity, biodegradability, and cost-effectiveness, several obstacles remain to be addressed in future clinical translation. First, the high activity of nanoFe makes its difficult for long-term storage and intravenous or oral application. Then, although the high reducibility of nanoFe makes it quickly quench ROS caused by inflammation, it tends to be transformed into Fe^2+^ after releasing electrons. Subsequently, Fe^2+^ can convert less reactive hydrogen peroxide into more ROS, which may be contrary to the original intention of the treatment [50]. Fe^2+^ or Fe^3+^ traversed from nanoFe may coordinate with biomolecules to some extent, resulting in adverse effects such as agglomeration. Interestingly, the inert modification of the nanoFe surface may slow down the rate of oxidation corrosion, thus enhancing its stability. In addition, the conversion of nanoFe into water-dispersible nanosulfur or nanoselenium can prevent additional ROS damage, which will be the focus of our future study. Nonetheless, the present study fully elucidates the value of nanoFe as a potential protection candidate against myocardial injury during sepsis.

## Conclusion

**Fig. 10:**
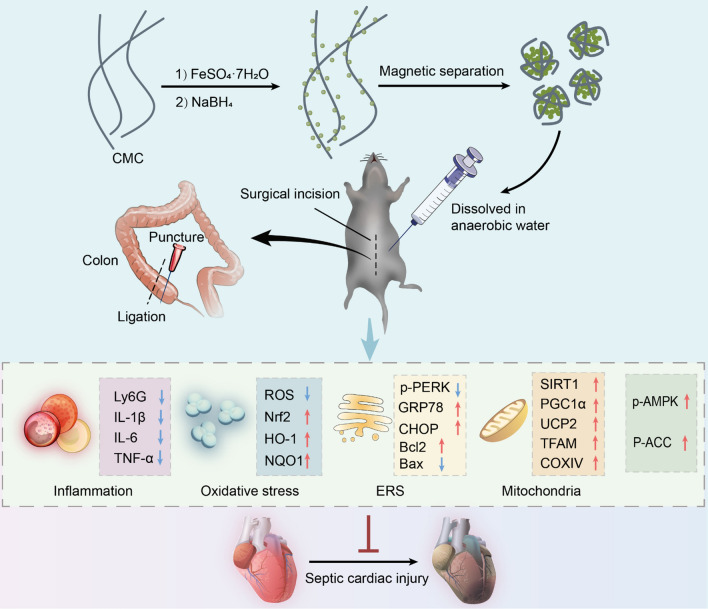
Proposed scheme depicting the mechanisms of nanoFe protecting against sepsis-induced cardiac injury

In conclusion, the current study demonstrates for the first time the beneficial role of nanoFe in sepsis and septic myocardial injury, primarily through attenuation of inflammation, inhibition of oxidative stress, improvement of mitochondrial function, regulation of ERS, inhibition of apoptosis, and activation of AMPK signaling pathways (Fig. 10). Next, we will further investigate the inert modification of nanoFe in sepsis to fully understand the role of nanoFe in sepsis and avoid the adverse effects of nanoFe per se.

## Methods

### Preparation of the nanoFe

Sodium CMC, NaBH_4_, and FeSO_4_·H_2_O were purchased from Aladdin Chemical Reagent Co., Ltd. (> 98%). Ultrapure water (18.2 MΩ cm^−1^) was obtained using a Millipore Milli-Q purification system and used throughout the experiments after purging with nitrogen gas for 30 min. FeSO_4_·H_2_O (1.00 g) was dissolved in 100 mL 0.5% sodium CMC aqueous solution under mechanical stirring. After 5 min, ice-cold NaBH_4_ aqueous solution (0.35 g, 20 mL) was added for 1 min. The mixture solution darkened quickly and was stirred for 30 min. Ultrapure water (5 × 50 mL) was used to wash the black sediment by magnetic separation and then dispersed in 0.5% CMC solution under ultrasound as a stock solution.

### Characterization

High-resolution TEM images were recorded on a JEOL-2100 electron microscope at 200 kV. Before analysis, the TEM samples were prepared by depositing 5 μL of the clarifying suspensions on a carbon film with a 230-mesh copper microgrid in the air at ambient temperature. XRD patterns were recorded from 10° to 90° at a speed of 0.1°·s^−1^ on a D8 ADVANCE (Bruker, Germany) X-ray diffractometer using Cu-Kα radiation (λ = 1.5418 Å) at 40 kV and 40 mA. Raman spectra were recorded on an InVia Qontor (Renishaw, UK) using a 532 nm laser at 50 mW. The surface chemistry of the nanoFe was investigated by XPS. An ESCALAB Xi + (Thermo Fisher Scientific, USA) spectrometer was used to acquire the survey and detailed spectra using a monochromatic Al Kα source (1486.6 eV at 150 W; beam size = 500 µm).

### Experimental design

NanoFe was dispersed in deoxygenized water and intraperitoneally injected into mice every two days three times in total before CLP surgery. Mice were randomly allocated into the following groups: mice injected with 1 mL/kg deoxygenization water and subjected to sham surgery; mice injected with 1 mL/kg deoxygenization water and then subjected to CLP surgery; and mice injected with nanoFe and then subjected to CLP surgery. In the third group, three groups of mice were injected with different concentrations of iron nanoparticles (5, 10 and 20 mg/kg) to observe the effect of nanoFe on the survival rate within 96 h of CLP. Then, 20 mg/kg was selected for further functional analysis, including cardiac function, routine blood, blood biochemical, and molecular parameters at 8 h post-CLP (Additional file 1: Fig. S4). For the CC experiment, in addition to the sham, CLP, and nanoFe + CLP groups, two additional groups were used: mice injected with 20 mg/kg nanoFe and 2.5 mg/kg CC (dissolved in DMSO) and then subjected to CLP surgery and mice injected with 2.5 mg/kg CC and then subjected to CLP surgery (Additional file 1: Fig. S4).

### Animal

Adult male Balb/c mice aged 8–10 weeks were obtained from the Airforce Medical University (Xi’an, China). Adult male Balbc mice aged 8–10 weeks and weighed 22–25 g were obtained from the Airforce Military Medical University (Xi’an, China). All animal experiment protocols (No. 2019008) were performed following the guidelines of the Animal Care and Use Committees at the Northwest University (Xi’an, China) and complied with the Guidelines for the Care and Use of Laboratory Animals (NIH Publication No. 85–23, revised 1996). Before the operation, all mice had free access to food and water and were bred at 26 ℃ in a 12 h light /12 h dark cycle. Cardiac function, blood routine, and blood biochemical parameters were detected at 8 h post CLP. Animals were euthanized at the endpoint, and hearts were removed for histological and molecular biological detection.

### CLP model

All mice were fasted for 8 h before the operation but had free access to water. Mice were anesthetized with 3% inhaled isoflurane. The abdominal hair of the mice was shaved off, and the abdominal skin was sterilized with 75% alcohol. The establishment of the CLP model mainly included two steps: A 1–2 cm midline laparotomy was performed to expose the cecum with the adjoining intestine. The cecum was tightly ligated at 1/3 of the site from its end with a blunted 14-gauge needle using 4–0 nylon suture, and double punctures of the cecal wall were performed with a 25 G needle (obtain a model with a minor injury, Additional file 1: Fig. S2A). A small droplet of feces was squeezed through the puncture site to ensure patency, and it was returned to the peritoneal cavity. Then, the incision of peritoneum, fasciae, abdominal musculature and skin was sutured with sterile 6–0 silk. All operated mice were resuscitated by injecting prewarmed normal saline (1 mL/100 g, 37 °C) subcutaneously. For a survival experiment, an aggravated CLP model was constructed (Additional file 1: Fig. S2B). The sepsis score was determined by two investigators after the induction of sepsis for 8 h by the murine sepsis score (MSS). Anal temperature was determined at 8 h post-CLP with an animal thermometer (Calvin Biotechnology Co., Ltd., Nanjing, Jiangsu, China).

### Detection of routine blood parameters and blood biochemical parameters

At 8 h post-CLP, 10 μL of blood was collected from the left eyeball in a strictly sterile procedure. The levels of WBCs, lymphocytes (LYMs), monocytes (MONs), granulocytes (RAAs), PLTs, and RBCs were detected by an automatic blood analyzer (XinRui Sunshine Technology Co., Ltd., KT-6200, Guangdong, China). Then, 150 μL of serum was collected from the blood. An automatic blood biochemistry analyzer (XinRui Technology Co., Ltd., XR210, Guangdong, China) was used to detect the levels of LDH, CK, AST, albumin (ALB), and blood urea nitrogen (BUN).

### Echocardiography evaluation

Echocardiography was performed using an animal-specific instrument (VisualSonics Vevo3100, VisualSonics, Toronto, ON, Canada) at 8 h post-CLP in the animals. Anesthesia was induced with 3% isoflurane and 1 L/min 100% oxygen in an induction chamber for 1–2 min. Once the animal lost its righting reflex, it was laid supine on a warm platform with its nose enveloped in a nose cone to keep the mouse anesthetized by 1.5% isoflurane, and M-mode images were recorded. The cardiac function parameters, including CO, SV, LVEDV, LVESV, left ventricular anterior wall thickness of the systole period (LVAWs), left ventricular anterior wall thickness of the diastole period (LVAWd), heart rate (HR), and LV mass, were calculated using Vevo LAB 3.0.0 software. All measurements were based on 3 consecutive cardiac cycles.

### Histological staining

The myocardium was fixed in 4% paraformaldehyde and sectioned at a thickness of 4–5 µm. The degree of cardiac fibrosis was examined by Masson staining (Servicebio, Co., Ltd, Wuhan, Hubei, China). Myocardial reactive oxygen species generation was examined by DHE staining (Beyotime Biotechnology, Shanghai, China). For immunostaining, paraffin-embedded slices were stained with the respective primary antibodies against Ly6G (1:200), NOX2 (1:200), IL-6 (1:200), and TNF-α (1:200), incubated with a secondary biotinylated anti-rabbit IgG, stained with 3,3’-diaminobenzidine (DAB), and imaged using a microscope (Invitrogen EVOS M5000, Thermo Fisher Scientific, Waltham, MA, USA). The above antibodies were all purchased from Servicebio Co., Ltd., Wuhan, Hubei, China. Quantitative analysis was performed by using Image-Pro Plus 6.0 software (Media Cybernetics, Silver Spring, USA).

### qRT-PCR analysis

Total RNA was extracted from cells using the TRIzolTM total RNA extraction kit (TAKARA BIO INC. Kusatsu, Shiga, Japan), and reverse transcription was performed using the Prime Script RT Master Mix (Hunan Accurate Biotechnology Co. Ltd. Hunan, China). Then TNF-α, IL-6, CXCL2, NLRP3, IL-1β, and Caspase-1 mRNA level is detected using quantitative real-time reverse transcriptase PCR analyses with SYBR Premix Ex Taq (Hunan Accurate Biotechnology Co. Ltd. Hunan, China). The forward primer sequence of TNF-α is 5′-ACTGAACTTCGGGGTGATCG-3′, the reverse primer sequence is 5′-TGGTGGTTTGCTACGACGTG-3′. The forward primer sequence of IL-6 is 5′-TCCGGAGAGGAGACTTCACA-3′, the reverse primer sequence is 5′-TGCCATTGCACAACTCTTTTCT-3′. The forward primer sequence of CXCL2 is 5′-CCACCAACCACCAGGCTACA-3′, the reverse primer sequence is 5′-CTGTAGCCTGGTGGTTGGT-3′. The forward primer sequence of NLRP3 is 5′-TCTACTCTATCAAGGACAGGAACG-3′, the reverse primer sequence is 5′-CCTTTCTCGGGCGGGTAAT-3′. The forward primer sequence of IL-1β is 5′-CCTTGTGCAAGTGTCTGAAGC-3′, the reverse primer sequence is 5′-AAGGGCTTGGAAGCAATCCT-3′. The forward primer sequence of caspase 1 is 5′-AGAACAGAACAAAGAAGATGGCACA-3′, the reverse primer sequence is 5′-GTGCCATCTTCTTTGTTCTGTTCTT-3′. The levels of the examined transcripts were compared to that of β-actin and normalized to the mean value of the controls.

### Western blot

Heart tissues were homogenized in RIPA buffer adding protease and phosphatase inhibitors (Beyotime Biotechnology, Shanghai, China). The protein concentration was determined by an Enhanced BCA Protein Assay Kit (Beyotime Biotechnology, Shanghai, China). Total protein extract was subjected to 10% (or 8%) SDS–PAGE and transferred onto PVDF membranes. The membranes was sealed with 5% skim milk for 1h, and then incubated with primary antibody at 4 °C overnight. All primary antibody information was as follow: antibodies against Ly6c, NQO1, ATF6, cleaved caspase 3, and TFAM (Santa Cruz Biotechnology, Dallas, TX, USA); HMGB1, F4/80, UCP2, and PGC-1α (Servicebio, Wuhan, China); Bcl2 and Nrf2 (Boster Biological Technology Co., Ltd., Inc. USA); PERK, CHOP, Bax, COXIV, ACC (Cell Signaling Technology, Inc, USA); IL-1β, p-PERK, SIRT1 (Bioss Biotechnology Co., Ltd, Beijing, China); AMPK, HO-1 (Abcam, Cambridge, United Kingdom). After incubation with the corresponding secondary antibodies (anti-mouse IgG, Cell Signaling Technology, Inc., USA; goat anti-rabbit IgG, Boster Biological Technology Co., Ltd., Inc. USA), fluorescent signal were detected using a MiNiChemi610 imaging system (SAGECREATION Co., Ltd, Beijing, China), and was quantified using ImageJ 1.8.0 software (National Institutes of Health, Bethesda, MD, USA).

### Bioinformatics analysis

#### Sample preparation

Total RNA was extracted from the tissue by TRIzol reagent (Invitrogen Life Technology Co., Ltd, USA). The RNA quality was checked by a Bioanalyzer 2200 (Agilent) and kept at − 80 °C. The RNA with RIN > 6.0 is suitable for the experiment.

#### cDNA library construction

The complementary DNA (cDNA) libraries were prepared by using the NEBNext™ Ultra Directional RNA Library Prep Kit, NEBNext Poly (A) mRNA Magnetic Isolation Module, and NEBNext Multiplex Oligos according to the manufacturer’s instructions. The products were purified and enriched by PCR to create the final cDNA libraries and quantified by Agilent 2200. The tagged cDNA libraries were pooled in equal ratios and used for 150 bp paired-end sequencing in a single lane of the Illumina *HiSeq X Ten.*

#### RNA sequencing mapping

Clean reads were obtained from the raw reads by removing the adaptor sequences, reads with > 5% ambiguous bases (noted as N), and low-quality reads containing more than 20% of bases with qualities of < 20. The clean reads were then aligned to the mouse genome (version: mm10 NCBI) using HISAT2 [51].

#### Gene expression

HTSeq-FPKM [52] was used to count gene and lncRNA counts, and the reads per kilobase per million mapped reads (RPKM) method was used to determine gene expression. Dif-gene-finder: After the significant analysis, *P value*, and false discovery rate (FDR) analysis, we applied the EBSeq algorithm [53] to filter the differentially expressed genes based on the following criteria in this reference [54].

#### GO analysis

GO analysis was performed to clarify the biological implications of unique genes [55]. We downloaded the GO annotations from NCBI (http://www.ncbi.nlm.nih.gov/), UniProt (http://www.uniprot.org/) and the Gene Ontology (http://www.geneontology.org/). Fisher’s exact test was applied to identify the significant GO categories, and FDR was used to correct the p values.

#### Pathway analysis

By pathway analysis, the significant pathways of the differentially expressed genes was determine based on the KEGG database. Fisher’s exact test was used to select the significant pathway, where *P* < 0.05 and FDR < 0.05 defined the threshold of significance [56].

### Statistical analysis

GraphPad Prism 9.0.0 statistic software was used to analyze the Data (LaJolla, CA, USA). All data are presented as the mean ± standard deviation (SD). For the difference analysis of experimental data, t test or one-way ANOVA was used depending on the experimental design. When ANOVA indicated significance, multiple comparisons were performed using Tukey’s HSD post hoc test. A value of *P* < 0.05 was considered significant.

## Supplementary Information

**Additional file 1: Figure S1.** The results on the acute toxicity of nanoFe. **A**, Weight change statistics. **B**, Representative images of H&E staining of mouse heart, liver, and kidney tissues. **Figure S2.** Establishment of mouse CLP models A and aggravated CLP models B. The cecum was tightly ligated at 1/3 site from its end using 4–0 nylon suture, and double punctures of the cecal wall were performed with a 25 G needle. For the aggravated CLP model, the cecum was tightly ligated at 2/3 site from its end. **Figure S3.** Additional echocardiographic data of nanoFe treatment on septic mice. **A,** Left ventricular end-systolic posterior wall thickness (LVPWs), left ventricular end-diastolic posterior wall thickness (LVPWd), heart rate (HR), and corrected left ventricular mass (LV mass) in the long axis view calculated 8 h after CLP. **B,** LVPWs, LVPWd, HR, and corrected LV mass in the short axis view calculated 8 h after CLP. ^*^P < 0.05, ^**^P < 0.01, ^***^P < 0.001, ^****^P < 0.0001 *vs.* Sham or *vs.* CLP; ns, non-significant. n = 6 for each group. **Figure S4.** A schematic illustration of the present study. **Table S1.** Peak area % mean value of nanoFe from XPS measurements. **Table S2.** Acute toxicity of nanoFe.

## Data Availability

All data and materials are available in the manuscript.

## Abbreviations

nanoFe: Zero-valent iron nanoparticles
CMC: Carboxymethylcellulose
CLP: Cecal ligation and puncture
ROS: Reactive oxygen species
HR-TEM: Transmission electron microscopy
XRD: X-ray diffraction pattern
XPS: X-ray photoelectron spectroscopy
H&E: Hematoxylin–eosin
WBC: White blood cells
PLT: Platelets
RBC: Red blood cells
LYM: Lymphocytes
MON: Monocyte
GRA: Granulocytes
LDH: Lactate dehydrogenase
CK: Creatine kinase
AST: Aspartate aminotransferase
BUN: Blood urea nitrogen
ALB: Albumin
CO: Cardiac output
SV: Stroke volume
LVEDV: Left ventricular diastolic volume
LVESV: Left ventricular systolic volume
LVAWs: Left ventricular anterior wall thickness of systole period
LVAWd: Left ventricular anterior wall thickness of diastole period
HR: Heart rate
ERS: Endoplasmic reticulum stress
TNF-α: Tumor necrosis factor-α
NLRP3: Nucleotide-binding domain leucine-rich repeat and pyrin domain containing receptor 3
Caspase-1: Cysteinyl aspartate specific proteinase
Nrf2: Nuclear factor-related factor 2
NQO1: NAD(P)H dehydrogenase quinone-1
HO-1: Heme oxygenase-1
PERK: PKR-like endoplasmic reticulum kinase
CHOP: C/EBP homologous protein
ATF6: Activating transcription factor 6
GRP78: Glucose-regulated protein 78
SIRT1: Silent information regulator 1
PGC-1α: Peroxisome proliferator-activated receptor coactivator-1α
TFAM: Mitochondrial transcription factor A
AMPK: AMP-activated protein kinase
ACC: Acetyl-coA carboxylase
DHE: Dihydroethidium
GO: Gene ontology
BP: Biological process
UPR: Unfolded protein response
Bcl2: B-cell lymphoma-2
Bax: Bcl-2-associated X
