# Supplementary material for: Protection of zero-valent iron nanoparticles against sepsis and septic heart failure
Source: J Nanobiotechnology. 2022 Sep 5;20:405. doi: 10.1186/s12951-022-01589-1 (PMC9444118; doi:10.1186/s12951-022-01589-1)
Supplement: Supplementary file 1 — Additional file 1: Figure S1. The results on the acute toxicity of nanoFe. A, Weight change statistics. B, Representative images of H&E staining of mouse heart, liver, and kidney tissues. Figure S2. Establishment of mouse CLP models A and aggravated CLP models B. The cecum was tightly ligated at 1/3 site from its end using 4–0 nylon suture, and double punctures of the cecal wall were performed with a 25 G needle. For the aggravated CLP model, the cecum was tightly ligated at 2/3 site from its end. Figure S3. Additional echocardiographic data of nanoFe treatment on septic mice. A, Left ventricular end-systolic posterior wall thickness (LVPWs), left ventricular end-diastolic posterior wall thickness (LVPWd), heart rate (HR), and corrected left ventricular mass (LV mass) in the long axis view calculated 8 h after CLP. B, LVPWs, LVPWd, HR, and corrected LV mass in the short axis view calculated 8 h after CLP. *P < 0.05, **P < 0.01, ***P < 0.001, ****P < 0.0001 vs. Sham or vs. CLP; ns, non-significant. n = 6 for each group. Figure S4. A schematic illustration of the present study. Table S1. Peak area % mean value of nanoFe from XPS measurements. Table S2. Acute toxicity of nanoFe. [file 12951_2022_1589_MOESM1_ESM.docx]

**Tables**

**Table S1.** Peak area % mean value of nanoFe from XPS measurements.

| The valence state of Fe in nanoFe | Content (%) |
| --- | --- |
| Fe(0) | 33.4 |
| Fe(II) | 35.5 |
| Fe(III) | 31.1 |

| Group | Dosage  (mg/kg) | Mice  number | Observation contents | | | | |  |
| --- | --- | --- | --- | --- | --- | --- | --- | --- |
|  |  |  | Appetite | Diarrhea | Hypnosi | Movement | Weight change | Mortality |
| Control (Female) | 0 | 2 | Normal | No | No | Normal | No | 0 |
| Control (Male) | 0 | 2 | Normal | No | No | Normal | No | 0 |
| NanoFe (Female) | 900 (Total) | 10 | Normal | No | No | Normal | No | 0 |
| NanoFe (Male) | 900  (Total) | 10 | Normal | No | No | Normal | No | 0 |

**Table S2.** Acute toxicity of nanoFe.

|  |
| --- |

**FIGURES**


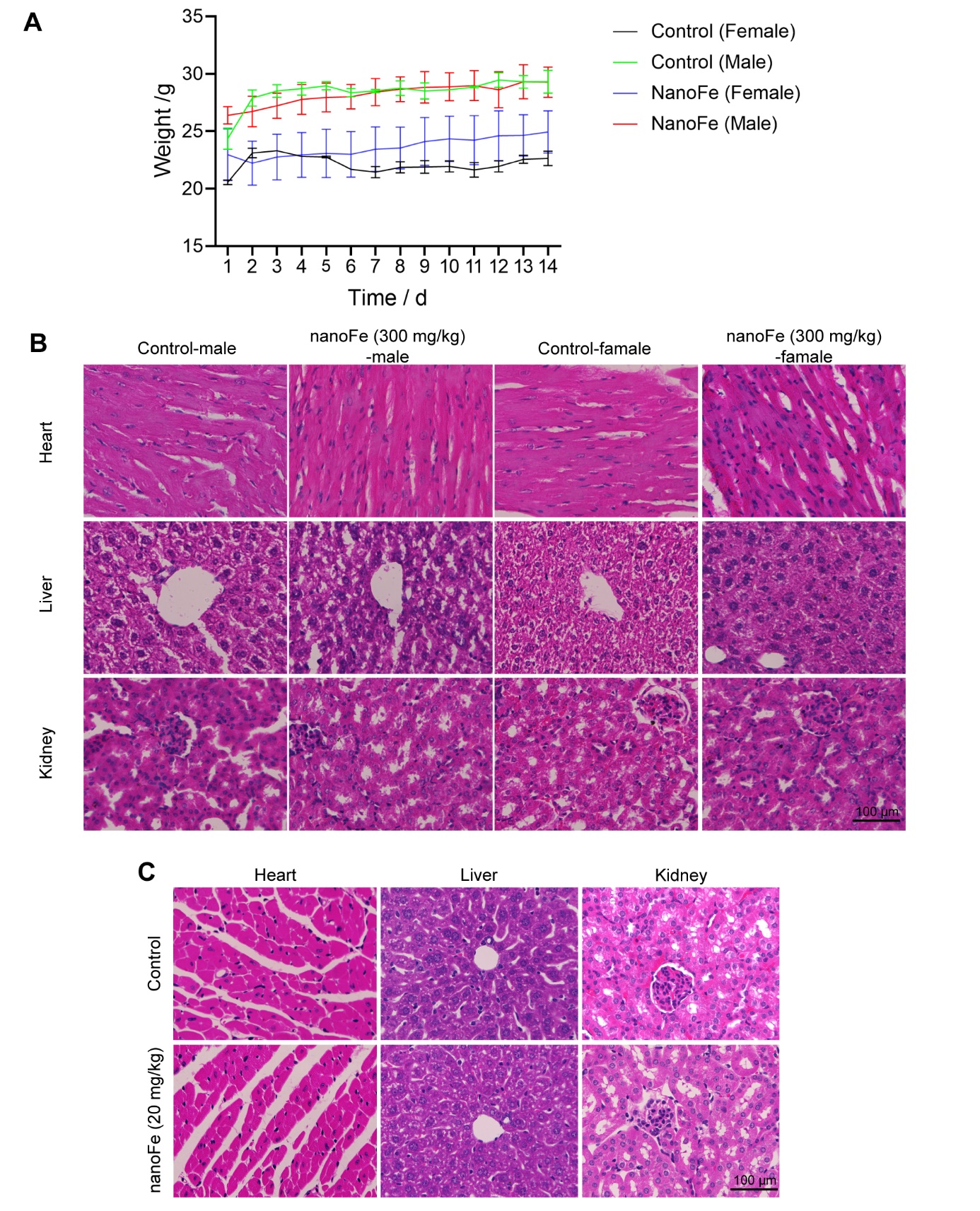


**Figure. S1.** The results on the acute toxicity of nanoFe. (**A**) Weight change statistics. (**B**) Representative images of H&E staining in the heart, liver, and kidney tissues of mice treated with 300 mg/kg nanoFe 3 times at an interval of 6 h. (**C**) Representative images of H&E staining in the heart, liver, and kidney tissues of mice treated with 20 mg/kg nanoFe 3 times at an interval of 1 d.


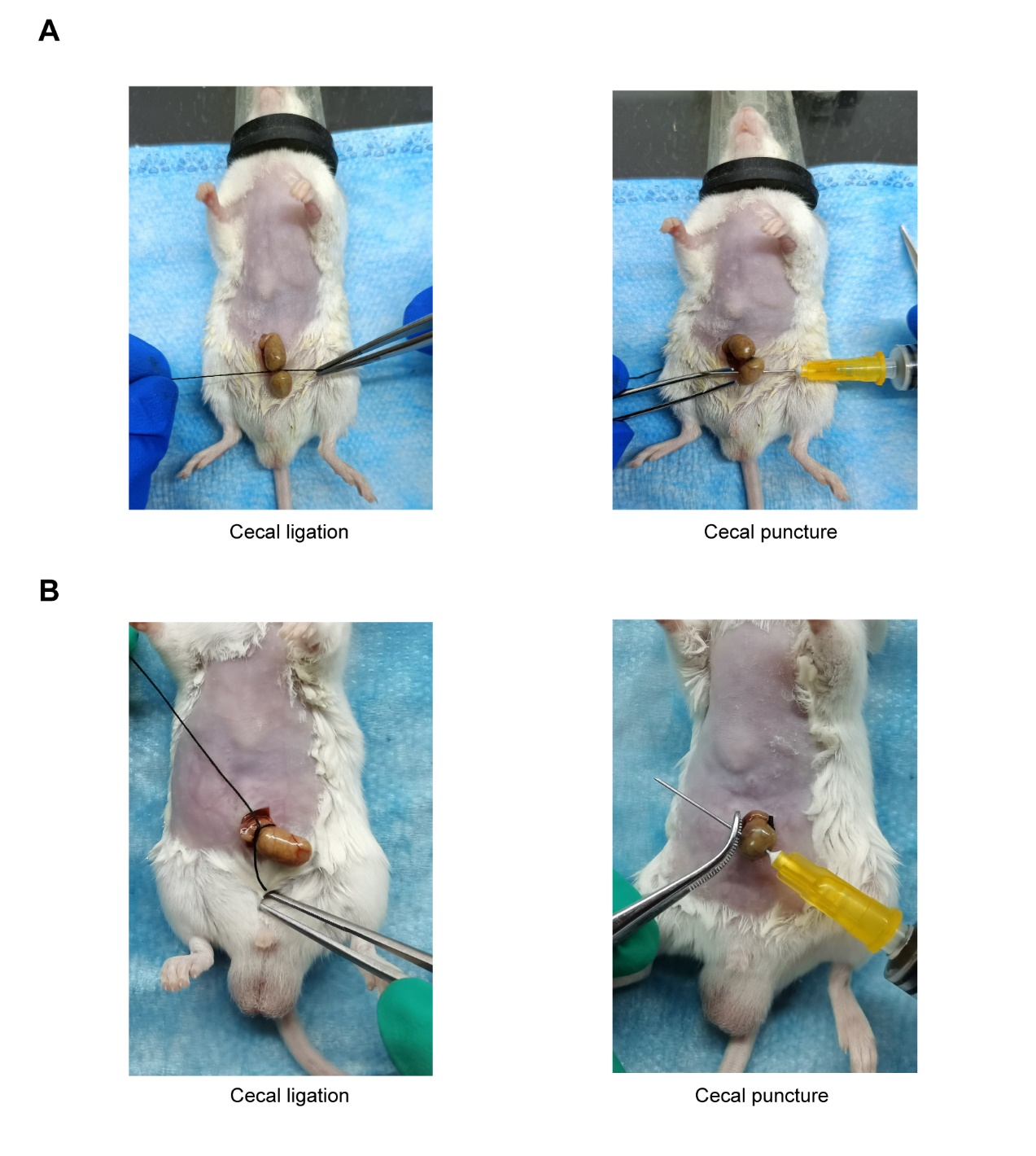


**Figure. S2.** Establishment of mouse CLP models A and aggravated CLP models B. The cecum was tightly ligated at 1/3 site from its end using 4-0 nylon suture, and double punctures of the cecal wall were performed with a 25 G needle. For the aggravated CLP model, the cecum was tightly ligated at 2/3 site from its end.

**
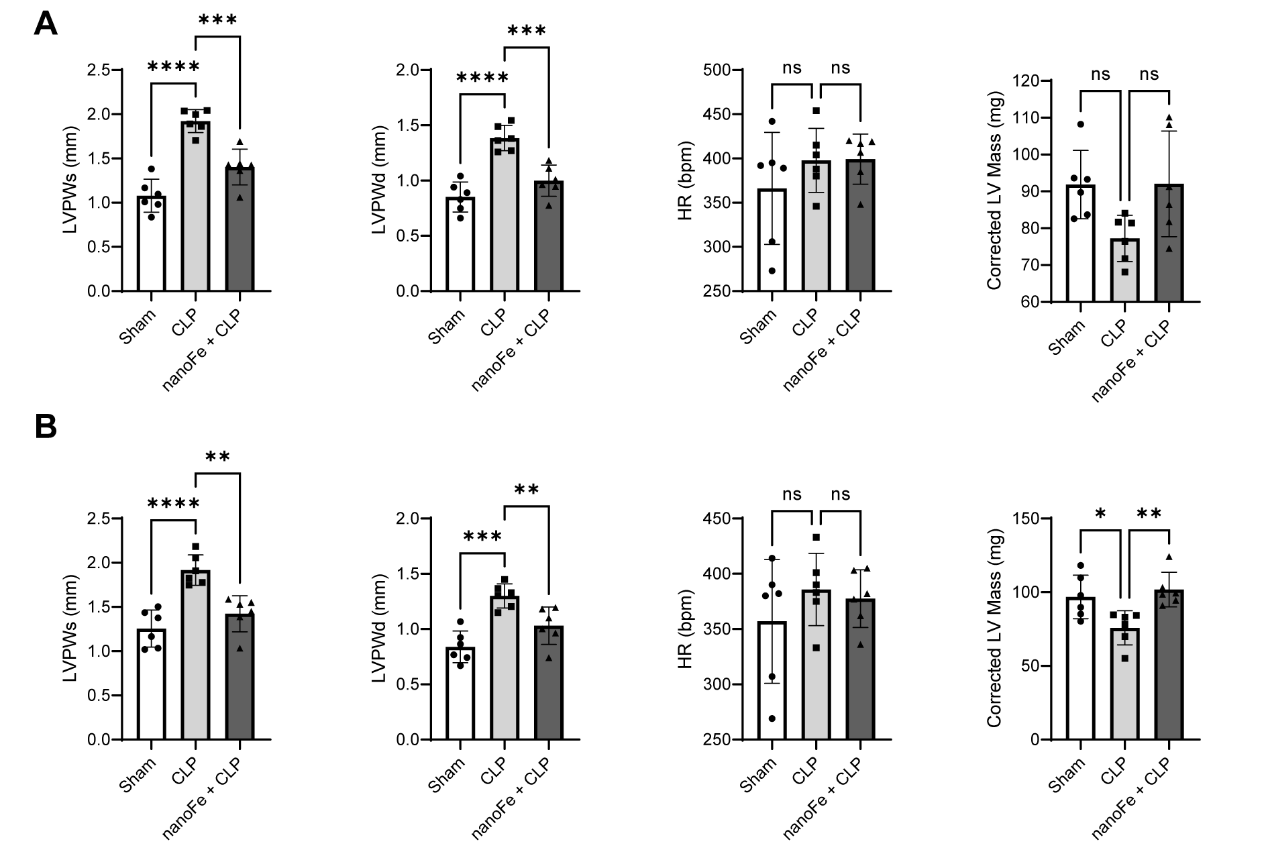
**

**Figure. S3.** Additional echocardiographic data of nanoFe treatment on septic mice. **A,** Left ventricular end-systolic posterior wall thickness (LVPWs), left ventricular end-diastolic posterior wall thickness (LVPWd), heart rate (HR), and corrected left ventricular mass (LV mass) in the long axis view calculated 8 h after CLP. **B,** LVPWs, LVPWd, HR, and corrected LV mass in the short axis view calculated 8 h after CLP. ^*^*P*<0.05, ^**^*P*<0.01, ^***^*P*<0.001, ^****^*P*<0.0001 *vs.* Sham or *vs.* CLP; ns, non-signiﬁcant. n=6 for each group.


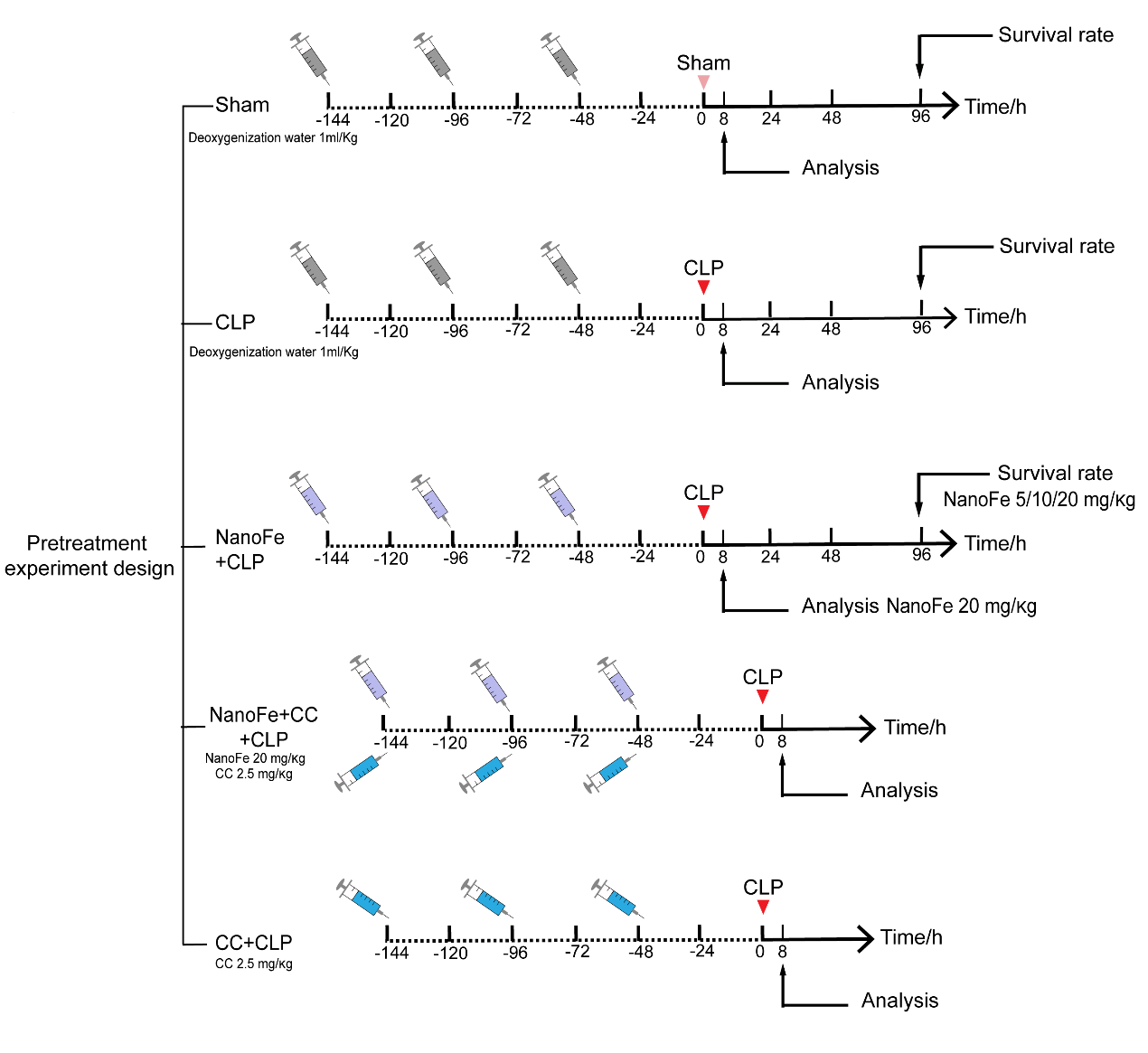


**Figure. S4.** A schematic illustration of the present study.
